# Supplementary material for: A high resolution 7-Tesla resting-state fMRI test-retest dataset with cognitive and physiological measures
Source: Sci Data. 2015 Jan 20;2:140054. doi: 10.1038/sdata.2014.54 (PMC4412153; doi:10.1038/sdata.2014.54)
Supplement: Supplementary Information [file sdata201454-s2.pdf]

# A high resolution 7-Tesla resting-state fMRI test-retest dataset with cognitive and physiological measures: Supplementary Materials

Krzysztof J. Gorgolewski[1], Natacha Mendes[1], Domenica Wilfling[2], Elisabeth Wladimirow[2], Claudine J. Gauthier[3,4], Tyler Bonnen[1], Florence J. M. Ruby[5], Robert Trampel[3], Pierre-Louis Bazin[3], Roberto Cozatl[6], Jonathan Smallwood[5], Daniel S. Margulies[1]

1 Max Planck Research Group for Neuroanatomy and Connectivity, Max Planck Institute for Human Cognitive and Brain Sciences, Leipzig, Germany
2 Department of Neurophysics, Max Planck Institute for Human Cognitive and Brain Sciences, Leipzig, Germany
3 Department of Neurology, Max Planck Institute for Human Cognitive and Brain Sciences, Leipzig, Germany
4 Concordia University/PERFORM Center, Montreal, Canada
5 Department of Psychology, University of York, York, United Kingdom
6 Databases and IT Group, Max Planck Institute for Human Cognitive and Brain Sciences, Leipzig, Germany

## Table of Contents

1. Table 1. Description of the columns in the questionnaires_and_blood_pressure.csv file. Page 2
2. Table 2. Description of the columns of the CCPT.csv file. Page 5
3. Table 3. Description of the columns of the mini_nyc_q.csv file. Page 6

**Table 1. Description of the columns in the questionnaires_and_blood_pressure.csv file.**

| **Column name** | **Description** | **German translation (if applicable)** |
|---|---|---|
| subject_id | Participant ID | n/a |
| session | Which visit the score corresponds to | n/a |
| panas_cheerful | PANAS-X item | fröhlich |
| panas_disgusted | PANAS-X item | angeekelt |
| panas_attentive | PANAS-X item | aufmerksam |
| panas_bashful | PANAS-X item | scheu |
| panas_sluggish | PANAS-X item | träge |
| panas_daring | PANAS-X item | wagemutig |
| panas_surprised | PANAS-X item | überrascht |
| panas_strong | PANAS-X item | stark |
| panas_scornful | PANAS-X item | voller Verachtung |
| panas_relaxed | PANAS-X item | entspannt |
| panas_irritable | PANAS-X item | reizbar |
| panas_delighted | PANAS-X item | erfreut |
| panas_inspired | PANAS-X item | angeregt |
| panas_fearless | PANAS-X item | furchtlos |
| panas_disgusted_with_self | PANAS-X item | vor mir selbst geekelt |
| panas_sad | PANAS-X item | traurig |
| panas_calm | PANAS-X item | ruhig |
| panas_afraid | PANAS-X item | ängstlich |
| panas_tired | PANAS-X item | müde |
| panas_amazed | PANAS-X item | verblüfft |
| panas_shaky | PANAS-X item | unsicher |
| panas_happy | PANAS-X item | glücklich |
| panas_timid | PANAS-X item | zaghaft |
| panas_alone | PANAS-X item | allein |
| panas_alert | PANAS-X item | hellwach |
| panas_upset | PANAS-X item | verärgert |
| panas_angry | PANAS-X item | wütend |
| panas_bold | PANAS-X item | kühn |
| panas_blue | PANAS-X item | trübsinnig |
| panas_shy | PANAS-X item | schüchtern |

| | | |
|---|---|---|
| panas_active | PANAS-X item | aktiv |
| panas_guilty | PANAS-X item | schuldig |
| panas_joyful | PANAS-X item | freudig |
| panas_nervous | PANAS-X item | nervös |
| panas_lonely | PANAS-X item | einsam |
| panas_sleepy | PANAS-X item | schläfrig |
| panas_excited | PANAS-X item | freudig erregt |
| panas_hostile | PANAS-X item | feindselig |
| panas_proud | PANAS-X item | stolz |
| panas_jittery | PANAS-X item | unruhig |
| panas_lively | PANAS-X item | lebhaft |
| panas_ashamed | PANAS-X item | beschämt |
| panas_at_ease | PANAS-X item | gelassen |
| panas_scared | PANAS-X item | verängstigt |
| panas_drowsy | PANAS-X item | dösig |
| panas_angry_at_self | PANAS-X item | über mich selbst verärgert |
| panas_enthusiastic | PANAS-X item | begeistert |
| panas_downhearted | PANAS-X item | niedergeschlagen |
| panas_sheepish | PANAS-X item | verlegen |
| panas_distressed | PANAS-X item | bedrückt |
| panas_blameworthy | PANAS-X item | tadelnswert |
| panas_determined | PANAS-X item | entschlossen |
| panas_frightened | PANAS-X item | furchtsam |
| panas_astonished | PANAS-X item | erstaunt |
| panas_interested | PANAS-X item | interessiert |
| panas_loathing | PANAS-X item | hasserfüllt |
| panas_confident | PANAS-X item | sebstsicher |
| panas_energetic | PANAS-X item | energiegeladen |
| panas_concentrating | PANAS-X item | konzentriert |
| panas_dissatisfied_with_self | PANAS-X item | mit mir selbst unzufrieden |
| hours_of_sleep_usually | "On average, how many hours do you sleep every night?" | "Wie viele Stunden schlafen Sie durchschnittlich pro Nacht?" |
| hours_of_sleep_last_night | "How many hours did you sleep last night?" | "Wie viele Stunden haben Sie letzte Nacht geschlafen?" |

| | | |
|---|---|---|
| vigilance | "How well rested do you feel right now? 1 (Extremely tired) - 9 (Perfectly well rested)" | "Wie ausgeruht fühlen Sie sich in diesem Moment? 1 (äußerst müde) - 9 (vollkommen ausgeruht)" |
| quality_of_sleep | "How well did you sleep last night? 1 (I slept terribly) - 9 (I slept very well)" | "Wie gut haben Sie letzte Nacht geschlafen? 1 (sehr schlecht) - 9 (sehr gut)" |
| thirst | "How well hydrated do you feel right now? 1 (Completely dehydrated) - 9 (Perfectly hydrated)" | "Wie durstig sind Sie in diesem Moment? 1 (äußerst durstig) - 9 (überhaupt nicht durstig)" |
| liters_of_water_daily | "On average, how much water (and other liquids) do you drink every day (in litres)?" | "Wie viel Wasser (oder andere Getränke) trinken Sie durchschnittlich pro Tag (in Liter)?" |
| relative_water_intake | "Comparing to other days did you drink more or less water today? 1 (I drank much less than usual) - 5 (I drank the same amount as usual) 9 (I drank much more than usual)" | "Haben Sie heute mehr oder weniger Wasser getrunken verglichen mit anderen Tagen? 1 (sehr viel weniger als gewöhnlich) - 5 (genauso viel wie gewöhnlich) - 9 (sehr viel mehr als gewöhnlich)" |
| caffeine_daily | "On average, how much caffeinated drinks (coffee, cola, club mate etc.) do you drink every day (in litres - one cup = 0.2 l)?" | "Wie viel koffeinhaltige Getränke (Kaffee, Cola, Club Mate, etc.) nehmen Sie durschnittlich pro Tag zu sich (in Liter: 1 Glas = 0,2 L)?" |
| relative_caffeine_intake | "Comparing to other days did you drink more or less coffee and other caffeinated drinks (coffee, cola, club mate etc.) today? 1 (I drank much less than usual) - 5 (I drank the same amount as usual) 9 (I drank much more than usual)" | "Haben Sie heute im Vergleich zu anderen Tagen mehr oder weniger koffeinhaltige Getränke (Kaffee, Cola, Club Mate, etc.) zu sich genommen? 1 (sehr viel weniger als gewöhnlich) - 5 (genauso viel wie gewöhnlich) - 9 (sehr viel mehr als gewöhnlich)" |
| systolic_blood_pressure_left | Systolic blood pressure measured on the left arm | n/a |
| diastolic_blood_pressure_left | Diastolic blood pressure measured on the left arm | n/a |
| pulse_left | Pulse measured on the left arm | n/a |
| systolic_blood_pressure_right | Systolic blood pressure measured on the right arm | n/a |
| diastolic_blood_pressure_right | Diastolic blood pressure measured on the right arm | n/a |
| pulse_right | Pulse measured on the right arm | n/a |

**Table 2. Description of the columns of the CCPT.csv file.**

| Column name | Description |
|---|---|
| subject_id | Participant ID |
| session | Which visit the score corresponds to |
| trial_number | One shape presentation is one trial |
| response | Subject response ("space" or "None") |
| response_time | Subject response in milliseconds measured from stimulus onset |
| trial_ISI | Interstimulus interval |
| trial_shape | Shape and colour of the presented stimulus |

**Table 3. Description of the columns of the mini_nyc_q.csv file.**

| Column name | Description | German translation (if applicable |
| --- | --- | --- |
| subject_id | Participant ID | n/a |
| session | Which visit the score corresponds to | n/a |
| timepoint | When the measurement was taken: 1 - after CCPT, 2 - after first whole-brain rs-fMRI, 3 - after second whole-brain rs-fMRI, 4 - after the prefrontal rs-fMRI | n/a |
| positive | "I thought about something positive" | "habe ich an etwas Positives gedacht." |
| negative | "I thought about something negative" | "habe ich an etwas Negatives gedacht." |
| future | "my thoughts involved future events" | "habe ich an zukünftige Ereignisse gedacht." |
| past | "my thoughts involved past events" | "habe ich an vergangene Ereignisse gedacht." |
| myself | "my thoughts involved myself" | "habe ich über mich selbst nachgedacht." |
| people | "my thoughts involved other people" | "habe ich an andere Menschen gedacht." |
| surroundings | "my thoughts involved my surroundings" | "habe ich über meine derzeitige Umgebung nachgedacht." |
| vigilance | "I was fully awake" | "war ich vollkommen wach." |
| images | "my thoughts were in the form of images" | "hatte ich Gedanken in Form von Bildern." |
| words | "my thoughts were in the form of words" | "hatte ich Gedanken in Form von Worten." |
| specific_vague | "my thoughts were more specific than vague" | "waren meine Gedanken eher spezifisch als vage." |
| intrusive | "my thoughts were intrusive" | "waren meine Gedanken aufdringlich/eindringlich." |
